# Titles of Specialists

**Published:** 1882-11

**Authors:** 


### Titles of Specialists.

The following "Report of the Judicial Council of the American Medical Association" forms a part of the By-Laws of the Michigan State Medical Society:

"The acceptance of the honorable title" (doctor) "is presumptive evidence to the community that the man accepting it is ready to attend practically to any and all duties which it implies. As all special practice is simply a self-imposed limitation of the duties implied in the general title of Doctor, it should be indicated, not by special or qualifying titles, such as Oculist, Gynæcologist, etc., nor by any positive setting forth of qualifications, but by a simple, honest notice, appended to the ordinary card of the general practitioner, saying, 'practice limited to diseases of the eye or ear,' or 'to diseases peculiar to women,' or 'to midwifery exclusively,' as the case may be."—*Maryland Medical Journal.*

